# A Novel Bipolar Polypectomy Snare Can Be an Alternative Choice for Endoscopic Resection

**DOI:** 10.3389/fmed.2020.619844

**Published:** 2021-01-20

**Authors:** Shengsen Chen, Danping Zhou, Rongwei Ruan, Jiangping Yu, Yandong Li, Yuanshun Liu, Shi Wang

**Affiliations:** Department of Endoscopy, Cancer Hospital of the University of Chinese Academy of Sciences (Zhejiang Cancer Hospital), Institute of Cancer and Basic Medicine (IBMC), Chinese Academy of Sciences, Hangzhou, Zhejiang, China

**Keywords:** endoscopic resection, monopolar snare, bipolar snare, colorectal polyp, alternative choice

## Abstract

**Objective:** Endoscopic resection (ER) is more difficult and has a higher rate of complications, such as perforation and bleeding. The aim of this study was to evaluate the safety and feasibility of a bipolar polypectomy snare for ER.

**Methods:** Initial ER procedures in live pigs were carried out. Then, a human feasibility study was performed in patients with colorectal polyps. Finally, the finite element method was used to evaluate the safety and effectiveness of the new bipolar snare.

**Results:** In the live animal model, there were no significant differences in wound size and cutting time between monopolar and bipolar groups. The histological results (histological scores) of the two groups in porcine experiments were almost the same except that the incision flatness of bipolar group was better than that of the monopolar group. Incidence of bleeding and perforation was similar between the two groups in pigs' and patients' study. At last, the finite element model showed that the vertical thermal damage depth produced by bipolar snare system was approximately 71–76% of that produced by monopolar snare system at the same power.

**Conclusions:** The novel bipolar snare is feasible in patients with colorectal polyps and can be an alternative choice for ERs.

## Introduction

Cancer of the digestive tract such as esophageal cancer, gastric cancer, and colorectal cancer is a major cause of morbidity and mortality worldwide (1). Performing endoscopy screening is an important way to decrease mortality of digestive tract cancer. Screening and therapeutic endoscopy enable early detection and removal of cancer in the digestive tract, which significantly reduce cancer-related mortality (2–4).

Therapeutic colonoscopy, including colon polypectomy and endoscopic mucosal resection (EMR), has recently been widely performed as an effective and less invasive treatment strategy (5, 6). This treatment, called “day surgery,” can be performed without hospitalization. However, even when small lesions are removed, this treatment still poses an unavoidable risk of complications, such as bleeding or perforation (7–9). Patients with endoscopic resection (ER) of colon polyps report a bleeding rate of 1.3–2% and a perforation rate of 0.1–0.3% (10–14). Although perforation rarely occurs, it can be more severe than bleeding (12, 15). The incidence of complications is low, but many patients undergo polypectomy each year, and the total number of complications is large.

Various methods have been introduced to reduce the frequency of complications associated with therapeutic endoscopy. A bipolar snare is an option for ER. For bipolar device, the current passes only through the tissue between the two electrodes placed closely (16, 17). Williams and de Peyer first reported on the use of bipolar snares as a safe technique for polyp removal in 1979 (18). Tucker et al. evaluated the energy required for monopolar and bipolar snares and tissue damage created by the two snares in a canine model (19). Their data indicated that the energy required for a bipolar snare was greatly reduced, and tissue damage was also reduced. Therefore, their research suggested that a bipolar snare decreased the occurrence of perforations. Saraya et al. reported that the perforation rate of ER with a bipolar snare was as low as 0.08%. In terms of perforation rates, they also found that a bipolar snare seemed to be at least as safe as a monopolar snare for ER of colorectal polyps (8).

We have developed a novel bipolar polyp snare (AG-5304-242523). The most important characteristic of this device is the return electrode, which is assembled on the outer side of the transparent cap at the end of the endoscope. This structure concentrates the current at the polyp by passing current from one section of the wire loop through the polyp to another section of the wire loop. Thus, electric current is localized to the tissue immediately surrounding the wire snare and does not pass through the patient to a distant return electrode, as in monopolar procedures. This should theoretically reduce the incidence of transmural burns, perforations, and postoperative hemorrhage. In this study, we evaluated the safety and feasibility of bipolar snare for removal of colorectal polyps.

## Methods

### Experimental Animals

Ten pigs, no limitation with sex, weighted 30–40 kg. Gateway Medical Innovation Center [animal use license no.: SYXK (Shanghai) 2015-0025] is responsible for purchasing experimental animals and abided by SOP (SOP300 experimental pig maintenance). The experimental animals were purchased from Qidong Longyu Technology Agricultural Development Co., Ltd. [license no.: SCXK (Su) 2018-0004] and Shanghai Jiagan Biotechnology Co., Ltd. [license no.: SCXK (Shanghai) 2015-0005]. All laboratory animals will be distinguished by a unique identification code printed on the ear tag or other suitable identification system.

### Evaluation in Animals

EMR using the device was evaluated in live porcine models. Under general anesthesia, healthy pigs weighing 30–40 kg and aged 1–3 months underwent EMR. EMR was performed in the esophagus, stomach, and colon. AEU-120B was used as the electrosurgical generator with power at 30 W. This study was divided into two time points (acute and chronic). A total of 10 experimental animals were used. The grouping of experimental animals follows the random principle. There were four pigs at the acute time point. Bipolar snare and monopolar snare were tested on these four pigs, and data were collected at the same time. The animals were euthanized and autopsied, and from which specimens were taken immediately after the surgery. The remaining six pigs at chronic time point were observed, and results were recorded on the 13th day. Bipolar snare and monopolar snare were respectively tested on each of three pigs, and data were collected. After the end of the experimental surgery, these pigs were resuscitated, fed, and observed. After the observation period, they were euthanized and autopsied, and specimens were taken. Specifically, animal experiment was evaluated in terms of *en bloc* resection rate, cutting time, bleeding, perforation, thermal damage, and histopathologic change.

### Patients

**Figure 1:**
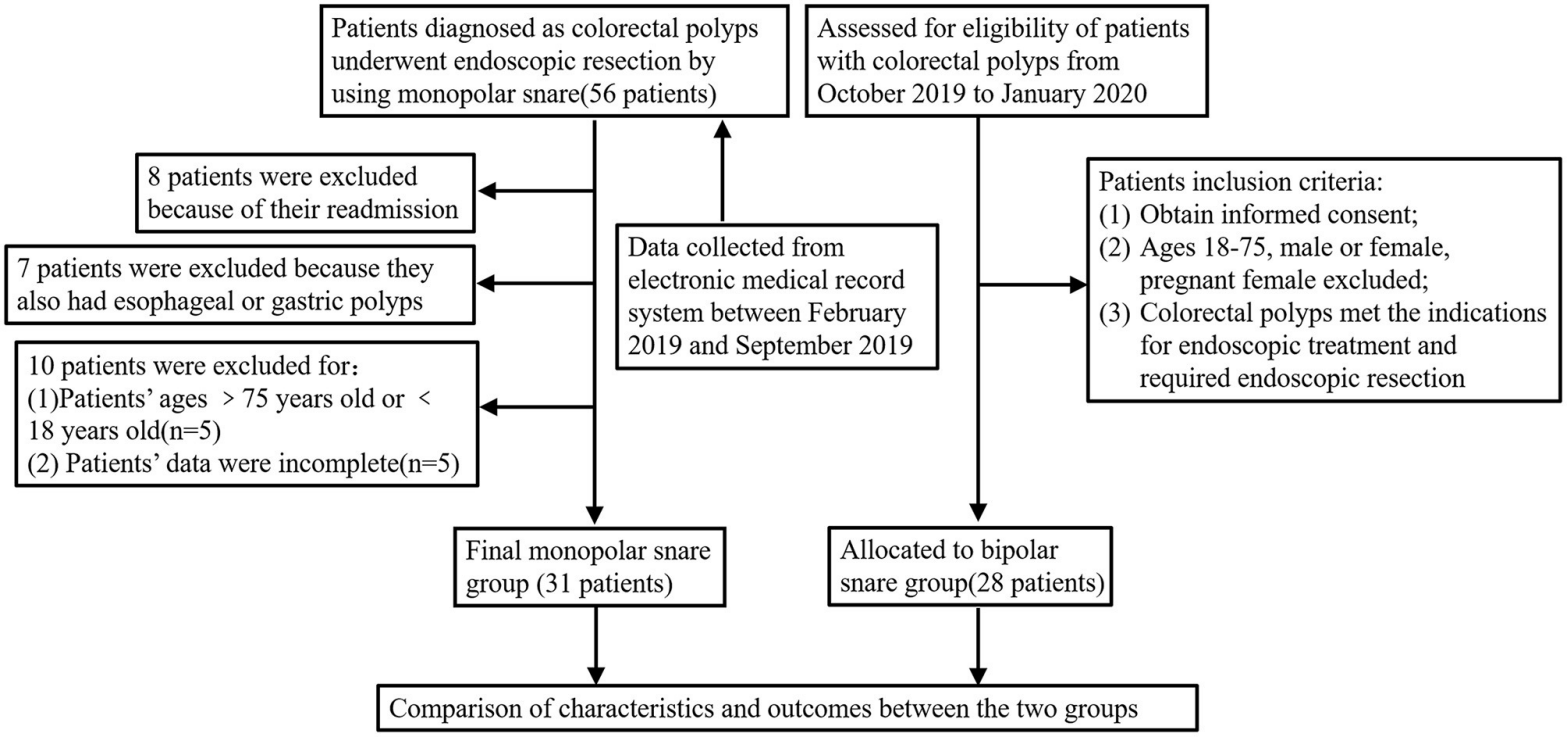
Flow diagram depicting the patient selection process.

Twenty-eight patients with colorectal polyps were enrolled into bipolar snare group according to the inclusion criteria (the detailed criteria can be seen in patient study protocol). Data of 31 patients in the monopolar snare group were collected from the electronic medical record system between February 2019 and September 2019. The detailed study design is summarized in Figure 1. If patients take anticoagulants, anticoagulants must be discontinued at least 1 week before surgery.

### Endoscopic Resection

We removed nearly all resectable lesions using the monopolar and bipolar snare by endoscope. An electrosurgery generator unit AEU-120B was used for ERs. The cutting mode was set at 30 W in autocut mode, and coagulation was performed at 60 W in forced coagulation mode. Lesions smaller than 10 mm in diameter were removed by polypectomy without submucosal injection. Lesions 10 mm in diameter or larger and those broad-based type were removed by the “EMR” method.

In addition, the pit pattern classification was a very important way to decide the procedure. Actually, we used the magnifying colonoscope to evaluate each case based on the pit pattern classification before ER. Furthermore, the endoscopic ultrasonography (EUS) was also used to evaluate the present or absent of submucosal deeply invasion. Therefore, we decided the procedure with polyp size, endoscopic diagnosis based on the pit pattern classification, and EUS result. Normal saline, 10% glycerol, and hyaluronic acid were used for submucosal injection. Clipping was performed for lesions showing immediate bleeding after ER, so no clips were used with prophylactic intent in this study. Three operators were extremely experienced in performing polypectomy and colorectal EMR using both monopolar and bipolar snares. They all had standard training and performed more than 1,000 EMR cases in total. Thus, our three endoscopists have good skills to perform the procedure safely.

### Thermodynamic Damage Model

The finite element method was used to establish a thermodynamic damage model of isolated pig liver tissue in high-frequency electrosurgical monopolar and bipolar systems. The pig liver tissue was selected to be 40 mm (length), 40 mm (width), and 10 mm (height). The electrode parts of the monopolar and bipolar snare have the same size and material (Supplementary Figure S1). The difference between the two snares is that the electrode of the monopolar snare needs to be connected to the ground plate to form a conductive circuit, whereas the electrode of the bipolar snare forms a conductive circuit with a return electrode assembled on the endoscope (Supplementary Figure S2).

This model uses a cuboid to simulate pig isolated liver tissue. It assumes that the heat flux at all other boundaries satisfies continuity. The electrode is positioned at the center of the upper surface of the cuboid, and its thermal properties are same as the surrounding area. In the monopolar system, the ground plate is attached to the lower surface of the tissue, and the snare is tightened along the convex portion of the tissue and current flows from the electrode through the tissue to the ground electrode plate to form a circuit (Supplementary Figures S2A,B). In the bipolar system, the return electrode is attached to the upper surface of the tissue, and the snare is tightened along the raised portion of the tissue, and current flows from the electrode through the tissue to the return electrode to form a circuit (Supplementary Figures S2C,D).

### Statistical Analysis

To compare the characteristics between the 2 groups, we used Kruskal–Wallis test or Student *t* test for continuous variables and Fisher exact test for dichotomous variables. All statistical analysis was performed using SPSS version 22.0 and GraphPad Prism version 7.0. All statistical tests were two-sided, and *P* < 0.05 was considered as statistically significant.

## Results

### Live Porcine Experiments

**Figure 2:**
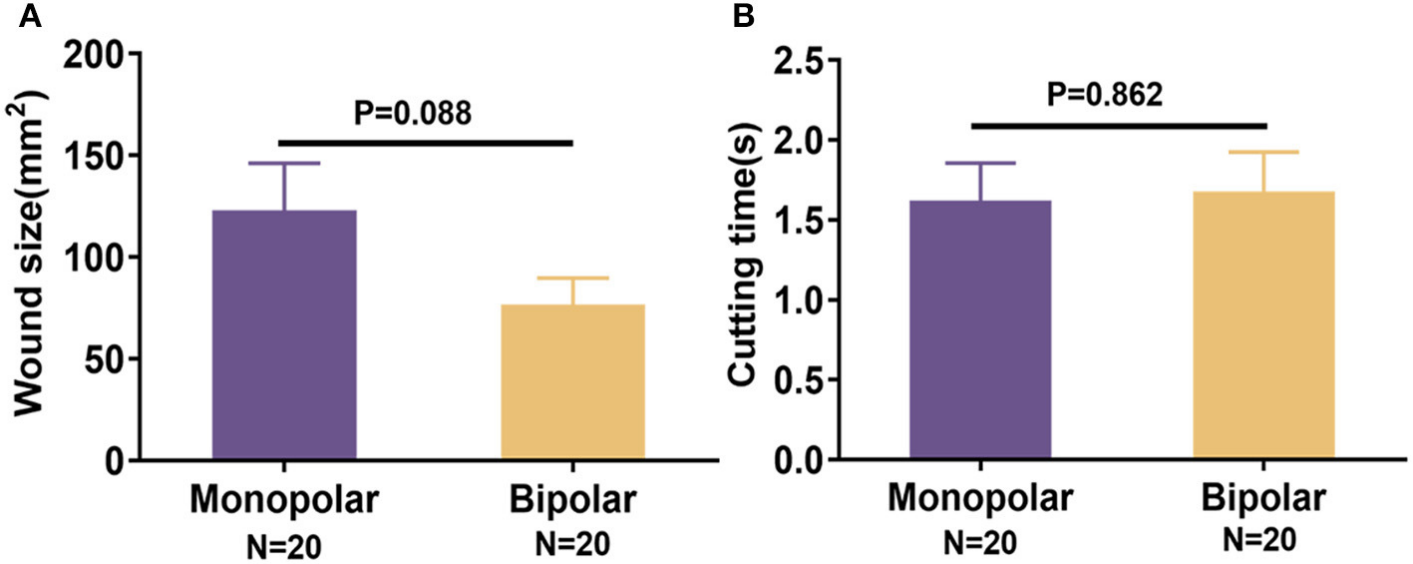
Comparison of wound size **(A)** and cutting time **(B)** in porcine experiments between monopolar group and bipolar group. Data were presented as means ± SD (*N* = total quantity of resection). *P*-values were calculated using the Student *t* test.

During the process of cutting mucosa of digestive tract, the average wound area of monopolar snare group was 123.1 ± 103.23 mm^2^, and the mean cutting time was 1.62 ± 1.06 s; the average wound area produced by bipolar snare was 76.68 ± 58.59 mm^2^, and the mean cutting time was 1.68 ± 1.10 s. There was no significant difference between the monopolar snare group and the bipolar snare group (Figure 2). The visible wounds of the digestive tract produced by the two snares were shown in Supplementary Figure 3. On the 13th day (chronic time point) after resection, the wounds of the digestive tract were almost healed in both groups (Supplementary Figure 4). The endoscopic mucosal *en bloc* resection rates in the monopolar and bipolar groups both were 100%. There was no immediate bleeding and perforation in the process of cutting mucosa between the monopolar group and the bipolar group. After 13 days of resection, pigs were reexamined by endoscope, and there was no delayed bleeding and perforation in the surgical wounds of monopolar and bipolar groups (Table 1).

**Table 1:** Ratio of *en bloc* resection, bleeding, and perforation in animal experiment.

**Parameter**	**Monopolar group**		**Bipolar group**	
	**(Event no./cutting no.)**	**%**	**(Event no./cutting no.)**	**%**
*En bloc* resection	20/20	100	20/20	100
Immediate bleeding	0/20	0	0/20	0
Delayed bleeding	0/9	0	0/9	0
Acute perforation	0/20	0	0/20	0
Chronic perforation	0/9	0	0/9	0

### Histological Change of the Animal Experiments

**Figure 3:**
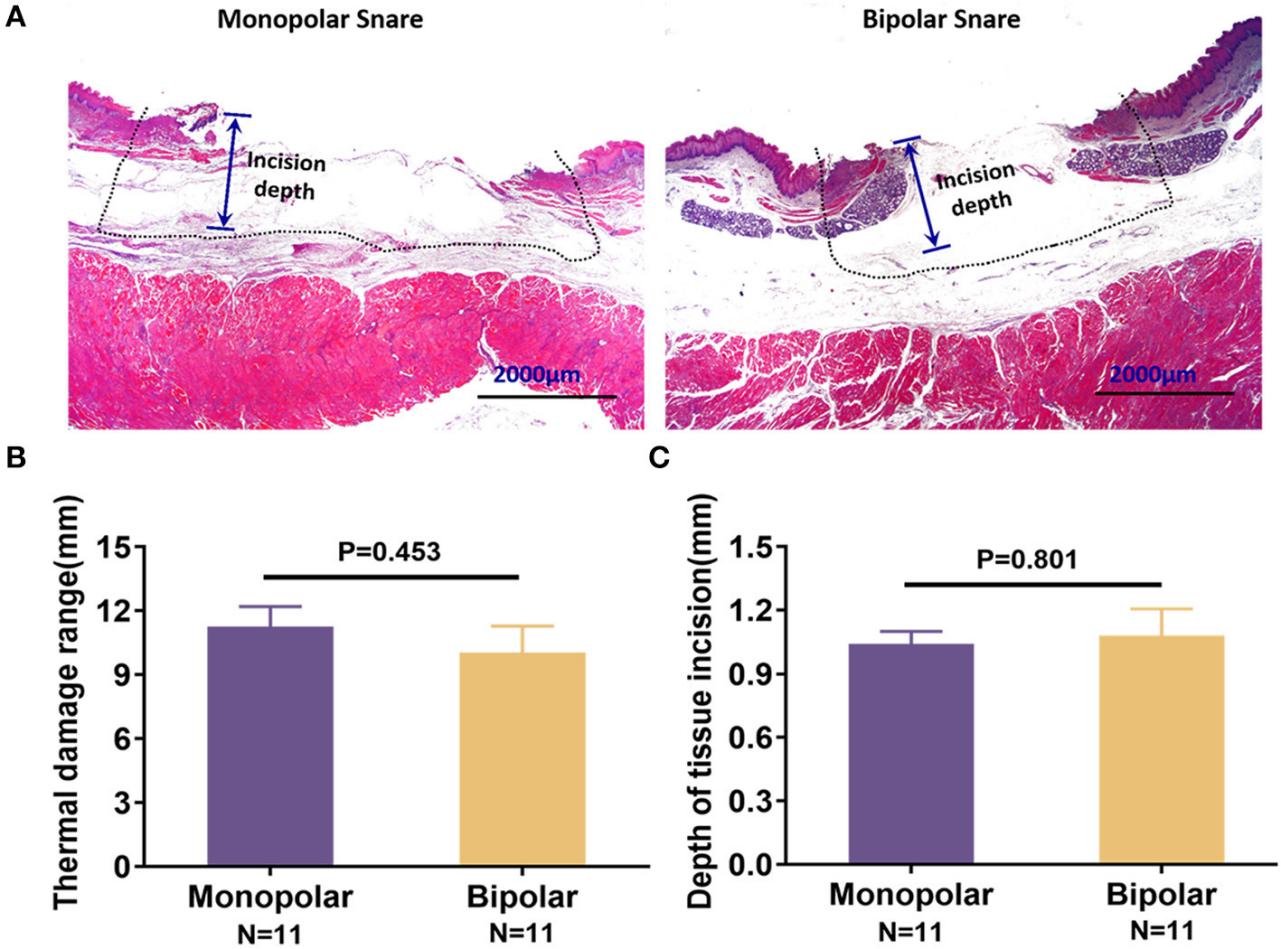
Acute injury of the digestive tract produced by the monopolar snare and bipolar snare during operation. **(A)** H&E staining of porcine esophagus mucosa removed with the two snares. The thermal damage range was indicted by dotted line. Scale bars: 2,000 μm. These pictures were captured by camera of a microscope. Comparison of thermal damage range **(B)** and incision depth **(C)** between the two snare groups. Thermal damage range and incision depth were calculated by software ImageJ. Data presented as means ± SD (*N* = total quantity of resection). Student *t* test was taken to do statistical analysis.

**Figure 4:**
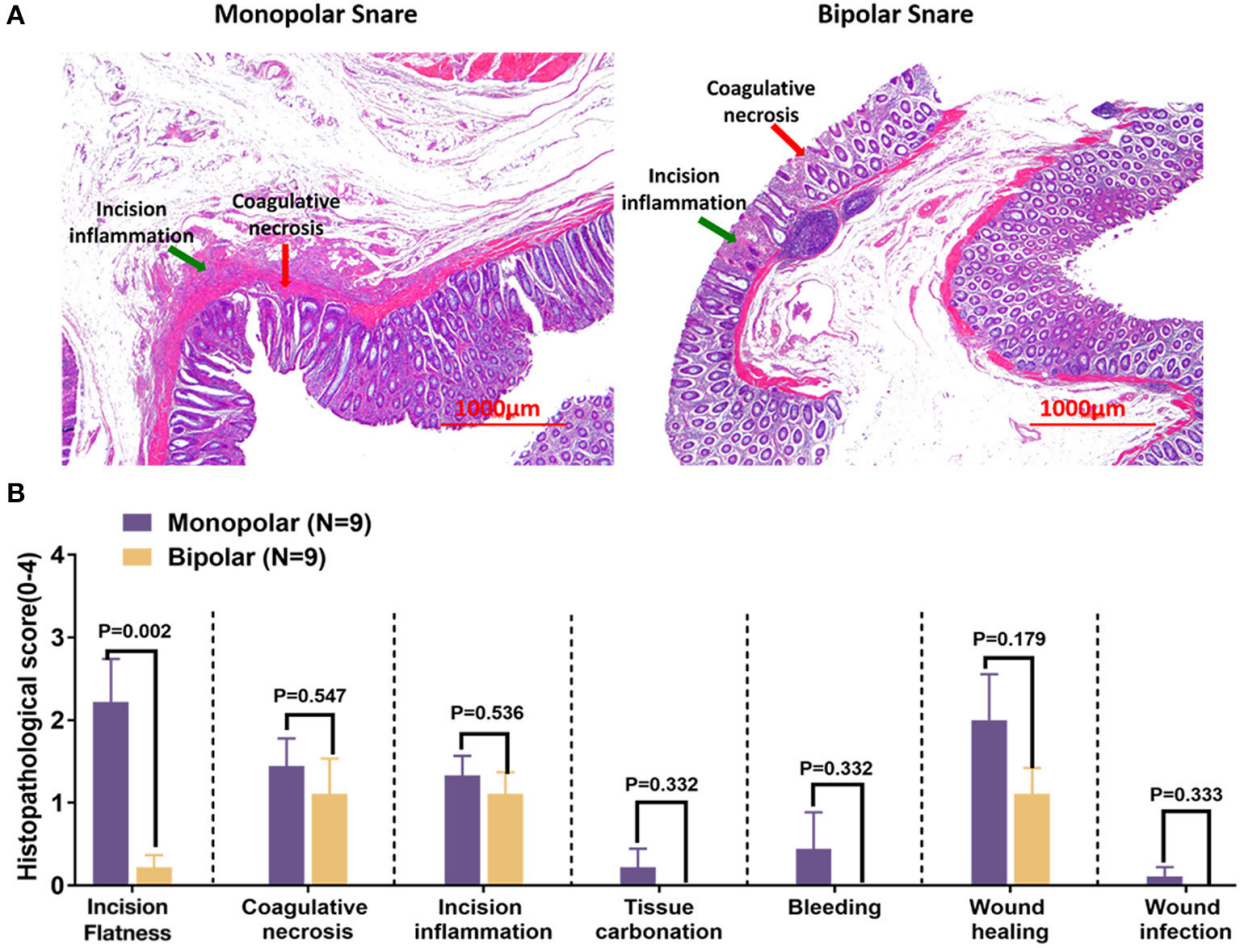
Histological changes of the digestive tract on day 13 after endoscopic resection by the monopolar snare and bipolar snare. **(A)** H&E staining of porcine rectal tissue removed by the two snares. Incision inflammation and coagulative necrosis were indicated by green arrow and red arrow, respectively. Scale bars: 1,000 μm. These pictures were captured by camera of microscope. **(B)** Histological scores of incision flatness, coagulative necrosis, incision inflammation, tissue carbonation, bleeding, wound healing, and wound infection were compared between monopolar and bipolar groups. The evaluation standards of these histological scores are summarized in Supplementary Tables 1–7. Data presented as means ± SD (*N* = total quantity of resection). Student *t* test was taken to do statistical analysis.

For acute time point, target lesion injury produced by monopolar and bipolar snares could be seen in Figure 3A, and the statistical results were expressed as mean ± SD as follows: thermal damage, monopolar group = 11.25 ± 3.19 mm, bipolar group = 10.03 ± 4.21 mm; incision depth, monopolar group = 1.04 ± 0.20 mm, bipolar group = 1.08 ± 0.42 mm. Thermal damage range and incision depth did not show any significant difference between the monopolar group and the bipolar group (Figures 3B,C). For chronic time point (on the 13th day after cutting), the histological change was shown in Figure 4A. Furthermore, at this time point, incision flatness histological score of the bipolar snare group was significantly lower than that of monopolar snare group (*P* = 0.002), whereas coagulative necrosis, incision inflammation, tissue carbonation, bleeding, wound healing, and wound infection all had no significant differences between the monopolar group and the bipolar group (Figure 4B).

### Endoscopic Resection in Patients

Fifty-nine patients were finally enrolled into our study: 31 in the monopolar snare group and 28 in the bipolar snare group. There was no significant difference in baseline characteristics such as age, gender, smoking, alcohol drinking, hypertension, and diabetes mellitus between the two groups (Table 2). As shown in Table 3, the colonic polyp characteristics were not different between the groups. The average number of polyps was 3.32 ± 1.99 in monopolar group and 3.25 ± 1.55 in bipolar group. The average polyp sizes were 1.29 ± 0.54 cm and 1.41 ± 0.53 cm. Regarding polyp morphology, granular polyps were most common in both groups (70.9 vs. 85.7%). For pit pattern classification and histological classification, the III-L type and adenoma were most common in both groups.

**Table 2:** Patients' characteristics comparison between monopolar group and bipolar group.

**Parameter**	**Monopolar snare (*n* = 31)**	**Bipolar snare (*n* = 28)**	P^*^
Age (years), mean ± SD	59.61 ± 7.91	62.71 ± 9.71	0.319
Male gender, *n* (%)	24 (77.4)	24 (85.7)	0.513
Smoking, *n* (%)	15 (48.4)	11 (39.3)	0.601
Alcohol drinking, *n* (%)	16 (51.6)	10 (35.7)	0.295
Hypertension, *n* (%)	9 (29)	7 (25)	0.776
Diabetes mellitus, *n* (%)	7 (22.6)	5 (17.9)	0.752
Length of in-hospital stay (days), mean ± SD	3.58 ± 0.76	3.79 ± 1.07	0.594
Preoperative CRP (mg/L), mean ± SD	1.66 ± 1.49	2.39 ± 3.61	0.554
Preoperative WBC count (10^9^/L), mean ± SD	6.02 ± 1.72	6.05 ± 1.39	0.976
Preoperative neutrophil count (10^9^/L), mean ± SD	3.83 ± 1.28	3.51 ± 1.11	0.191
Preoperative neutrophil percentage (%), mean ± SD	63.36 ± 7.20	62.06 ± 9.51	0.554

*SD, standard deviation; CRP, C-reactive protein; WBC, white blood cell*.

* *Categorical variables: Fisher exact test, continuous variables: Kruskal–Wallis test*.

**Table 3:** Characteristics of colorectal polyps in monopolar group and bipolar group.

**Parameter**	**Monopolar snare (*n* = 31)**	**Bipolar snare (*n* = 28)**	P*
Polyp number, mean ± SD	3.32 ± 1.99	3.25 ± 1.55	0.920
Polyp maximum size (cm),mean ± SD	1.29 ± 0.54	1.41 ± 0.53	0.271
**Morphology**, ***n*** **(%)**			0.379
Granular	22 (70.9)	24 (85.7)	
Non-granular	6 (19.3)	3 (10.71)	
Mixed	3 (9.7)	1 (3.6)	
**Pit pattern classification**, ***n*** **(%)**			0.830
I	0 (0)	0 (0)	
II	2 (6.5)	1 (3.6)	
III-L	18 (58.1)	17 (60.7)	
III-S	3 (9.7)	2 (7.1)	
IV	7 (22.6)	8 (28.6)	
V	1 (3.2)	0 (0)	
**Histopathology**, ***n*** **(%)**			0.966
Hyperplastic polyp	2 (6.5)	1 (3.6)	
Adenoma	24 (77.4)	22 (78.6)	
High-grade adenoma	4 (12.9)	4 (14.3)	
Adenocarcinoma	1 (3.2)	1 (3.6)	

SD, standard deviation. P*categorical variables—Fisher exact test, continuous variables—Kruskal–Wallis test.

Table 4 shows ER treatment outcomes and adverse events between two groups. The percentage of patients who underwent EMR was not statistically different in the two groups. All patients underwent *en bloc* resection in monopolar and bipolar groups. Regarding adverse events, there were no significant differences in the incidence of immediate bleeding or delayed bleeding. Perforation did not occur in both groups. Additionally, postoperative C-reactive protein, white blood cell count, neutrophil count, and neutrophil percentage were not statistically different between the monopolar group and the bipolar group.

**Table 4:** Outcomes and adverse events of colorectal endoscopic resection in monopolar group and bipolar group.

**Parameter**	**Monopolar snare (*n* = 31)**	**Bipolar snare (*n* = 28)**	P^*^
*En bloc* resection, *n* (%)	31 (100)	28 (100)	NA
**Procedure**, ***n*** **(%)**			0.604
Polypectomy	16 (51.6)	12 (42.9)	
EMR	15 (48.4)	16 (57.1)	
**Adverse events**, ***n*** **(%)**			
Immediate bleeding	0 (0)	0 (0)	NA
Delayed bleeding	1 (3.2)	3 (10.7)	0.337
Perforation	0 (0)	0 (0)	NA
Postoperative CRP (mg/L), mean ± SD	6.27 ± 6.94	5.03 ± 6.40	0.462
Postoperative WBC count (10^9^/L), mean ± SD	5.44 ± 1.48	5.49 ± 1.18	0.982
Postoperative neutrophil count (10^9^/L), mean ± SD	3.48 ± 1.07	3.38 ± 0.97	0.698
Postoperative neutrophil percentage (%), mean ± SD	63.84 ± 5.88	61.40 ± 8.51	0.163

*SD, standard deviation; CRP, C-reactive protein; WBC, white blood cell; NA, not available*.

* *Categorical variables: Fisher exact test, continuous variables: Kruskal–Wallis test*.

*Immediate bleeding defined as hemorrhage during the procedure. Delayed bleeding defined as bleeding that occurred at least 1 h after the procedure*.

### Finite Element Analysis of Two Electrosurgical Snares

**Figure 5:**
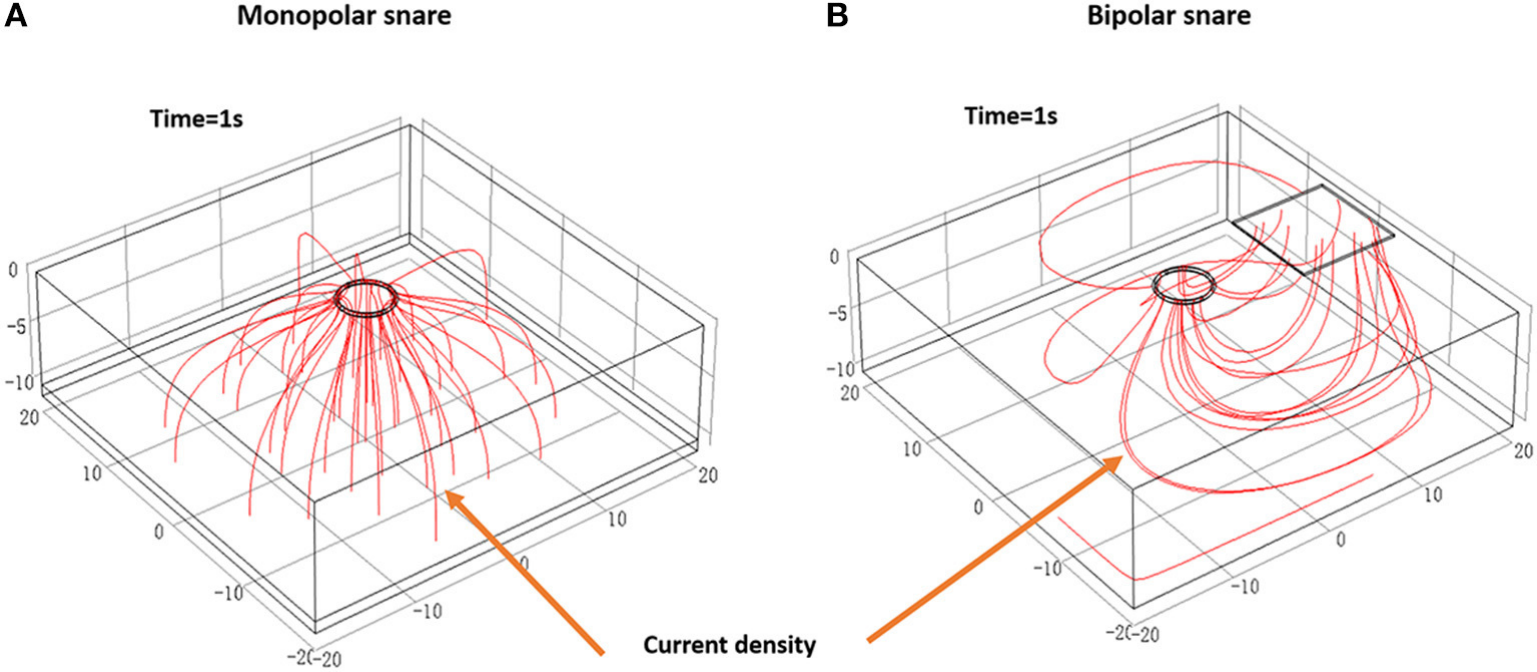
Potential distribution and current density streamline diagram of monopolar **(A)** and bipolar **(B)** electrosurgical systems. These figures were drawn by using software COMSOL Multiphysics (COMSOL Inc.), version 5.4 (https://cn.comsol.com/company).

**Figure 6:**
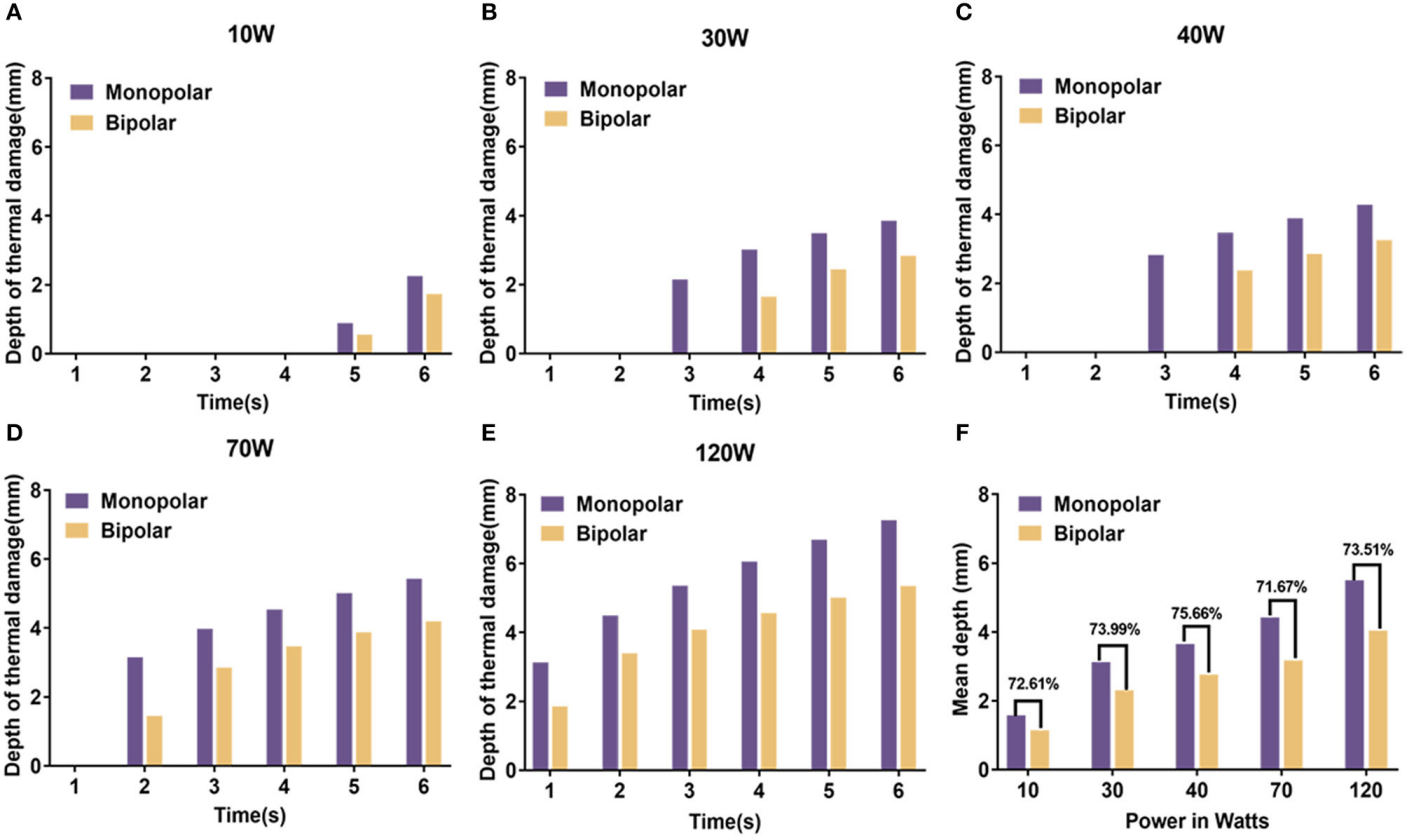
The vertical thermal damage depth analyzed by finite element method at power of 10 W **(A)**, 30 W **(B)**, 40 W **(C)**, 70 W **(D)**, and 120 W **(E)** with different time between monopolar snare and bipolar snare. **(F)** The ratio of the average vertical thermal damage depth between monopolar snare and bipolar snare at different powers. Because these data were collected from each individual case, statistical analysis cannot be done.

According to the short-term transient analysis of 1 s, the potential distribution and current density streamlines of the two electrosurgical snare systems are shown in Figure 5. We can see that the current flow direction of the monopolar snare is vertically downward from the electrode surface through the whole layer of tissue, and the current density in the central area is the largest, which gradually decreases outward. The current of bipolar snare flows horizontally from the electrode surface to the return electrode, and the current density in the surface is the largest, which gradually decreases downward. Then, we measured the vertical thermal damage depth of the area with its temperature >43°C at different power with different time (Figure 6). At power of 10, 30, 40, 70, and 120 W, the average vertical thermal damage depth of monopolar snare was 1.57, 3.12, 3.65, 4.42, and 5.50 mm, whereas the average vertical thermal damage depth of bipolar snare was 1.14, 2.31, 2.76, 3.17, and 4.04 mm. Under the same power condition, the vertical thermal damage depth produced by the bipolar snare system was approximately 71–76% of that produced by the monopolar snare system (Figure 6F).

## Discussion

Colorectal polyp ER is a form of minimally invasive surgery and is widely used (6). Almost all complications of ER with monopolar snares have been previously reported (20–22). A monopolar snare has only one electrode, and the other electrode (grounding pad) is located on the surface of the human body. Current leaves the snare and passes through the human tissue to the grounding pad and then flows back to the high-frequency generator to complete the circuit (17). The impedance encountered by a large area of current conducted in the patient's body can cause thermal damage to the tissue (17). Perforation rates of polypectomy and EMR by using a monopolar snare are, respectively, 0–0.1% (20, 21) and 0.4–1.5% (22, 23).

Use of a bipolar instrument is one way to reduce complications during therapeutic endoscopy.

A bipolar snare has active and return electrodes and does not need a grounding pad. Current leaves the active electrode, passes through only a small area of tissue, and then returns to the high-frequency generator via the return electrode of the bipolar snare (17–19). The current is only limited between the two electrodes, and the contact area between current and tissue is small. Within the controllable range of the surgical field, vertical thermal damage to the tissue is reduced. Thus, this device theoretically minimizes the degree of tissue destruction. In addition, bipolar snare is more suitable for patients with implantable medical devices than monopolar snare. The current of the monopolar snare is transmitted in a large area in the patient's body, and the electrical signal easily interferes with the normal operation of the implanted medical device. If the artificial pacemaker or defibrillator is interfered by electrical signal, then severe arrhythmia will come out. However, the bipolar snare current flow only localized in a small area between the two electrodes, and the probability of accessing the implanted medical device is very low (24, 25).

This study is significant because it evaluated a new electrosurgical bipolar snare for ER across a wide range of conditions from animal experiments to the human feasibility study. Pigs and patients did not experience any serious adverse events during or after ER, and electrical stability, durability, and effectiveness of the device were confirmed. The perforation was not found in the monopolar group and the bipolar group. Also, the incidence of immediate and delayed bleeding showed no significant difference between these two groups (Tables 1, 4). Because the perforation and bleeding rates appeared to be similar between the monopolar group and the bipolar group, we suggested that the bipolar snare was at least as safe as the monopolar snare for endoscopic removal of colorectal polyps. The *en bloc* resection rates, wound size, thermal damage range, and incision depth were almost the same in both groups (Table 1, Figures 2A, 3B,C).

What is more important was that we confirmed that cutting time of the new bipolar device was not obviously different from that of monopolar device in digestive tract mucosal ER surgery with the same wound size (Figure 2B), which meant that the cutting speed of our new bipolar snare was comparable to that of a monopolar snare. The reason that the cutting speed improved is that we assembled the return electrode on the end of the endoscope, which increased the contact area between return electrode and tissue, and reduced the resistance, thus increasing the current density and cutting efficiency under the same voltage. On the 13th day after operation, histopathologic results of the porcine study showed that incision flatness of the bipolar snare group was better than that of the monopolar snare group, whereas coagulative necrosis, incision inflammation, tissue carbonation, bleeding, wound healing, and wound infection all were not significantly different between the monopolar group and the bipolar group (Figure 4B). Therefore, we demonstrated that the bipolar snare was at least not inferior to monopolar snare for endoscopic removal of colorectal polyps based on the results of porcine and patients' study between the monopolar group and the bipolar group.

The accuracy of the results of this study may be affected by the individual difference (peristaltic frequency of digestive tract, thickness of mucosa, location and density of blood vessels) and surgical operating factors (operating experience and technique). Thus, in order to reduce external interference, we also used finite element method to evaluate the safety and effectiveness of the bipolar snare and found that the current flow of a monopolar snare was vertical (Figure 5A), whereas the current flow of the bipolar device was horizontal (Figure 5B), and the depth of vertical thermal damage to liver tissue produced by the bipolar snare system was approximately 71–76% of that produced by the monopolar snare system at the same power (Figure 6F). Because of small theoretical thermal damage, the use of bipolar snare has a theoretical lower risk of perforation than the use of monopolar snare.

In conclusion, the novel bipolar snare has similar cutting efficiency with monopolar snare. The sample size in this study was small, and the results were also affected by many factors such as patients' differences, so we cannot statistically evaluate which snare is safer. We only confirmed that our novel bipolar snare was not inferior to the monopolar snare. However, results of finite element analysis showed that the bipolar snare tented to be safer than the monopolar snare. Additionally, use of bipolar device may avoid situations when a special patient cannot use a monopolar knife, such as the patient with cardiac pacemaker implantation. Therefore, we think that the novel bipolar snare can be an alternative choice for ER. But a larger analysis of human data samples for comparing monopolar and bipolar instruments is needed in the future.

## Data Availability Statement

The original contributions presented in this study are included in the article/Supplementary Materials, further inquiries can be directed to the corresponding author/s.

## Ethics Statement

The studies involving human participants were reviewed and approved by Ethics committee of Zhejiang Cancer Hospital. The patients/participants provided their written informed consent to participate in this study. The animal study was reviewed and approved by Ethics committee of Zhejiang Cancer Hospital.

## Author Contributions

SW conceived the idea and designed experiments. DZ, YuL, and YaL collected data. SC analyzed the data and drafted the manuscript. SW, JY, and RR performed the endoscopic resection. All authors contributed to the article and approved the submitted version.

## Conflict of Interest

The authors declare that the research was conducted in the absence of any commercial or financial relationships that could be construed as a potential conflict of interest.
