# Supplementary material for: A Novel Bipolar Polypectomy Snare Can Be an Alternative Choice for Endoscopic Resection
Source: Front Med (Lausanne). 2021 Jan 20;7:619844. doi: 10.3389/fmed.2020.619844 (PMC7855578; doi:10.3389/fmed.2020.619844)
Supplement: Supplementary file 1 [file Data_Sheet_1.DOCX]

**A novel bipolar** **polypectomy snare can be an alternative choice for endoscopic resection**

Shengsen Chen^1^, Danping Zhou^1^, Rongwei Ruan^1^, Jiangping Yu, Yandong Li, Yuanshun Liu, Shi Wang*

Department of Endoscopy, Cancer Hospital of the University of Chinese Academy of Sciences(Zhejiang Cancer Hospital), Institute of Cancer and Basic Medicine(IBMC), Chinese Academy of Sciences, Hangzhou 310022, Zhejiang, China.

*Corresponding author: Shi Wang. Department of Endoscopy, Cancer Hospital of the University of Chinese Academy of Sciences(Zhejiang Cancer Hospital), Institute of Cancer and Basic Medicine(IBMC), Chinese Academy of Sciences, Hangzhou 310022, Zhejiang, China.

E-mail: wangshi@zjcc.org.cn, telephone number: (+86) +86-571-88122277, fax number: +86-571-88122277.

^1^ Shengsen Chen, Danping Zhou and Rongwei Ruan contributed equally to this work.

**The Histological evaluation standard**

| Supplementary Table S1. Histopathological score of bleeding | |
| --- | --- |
| Score | Features |
| 0 | No bleeding |
| 1 | Occasional bleeding around the tissue |
| 2 | Small but significant bleeding around the tissue |
| 3 | Significant large bleeding area around the tissue |
| 4 | Massive bleeding around the tissue |

| Supplementary Table S2. Histopathological score of wound infection | |
| --- | --- |
| Score | Features |
| 0 | No infection, no bacterial colonization |
| 1 | Minor infection |
| 2 | Moderate infection |
| 3 | Moderate to severe infection |
| 4 | Severe infection |

| Supplementary Table S3. Histopathological score of incision inflammation | |
| --- | --- |
| Score | Features |
| 0 | No inflammatory cells around the incision |
| 1 | Small amount of inflammatory cell infiltration around the incision |
| 2 | Mild dense inflammatory cell infiltration around the incision |
| 3 | Moderate or above dense inflammatory cell infiltration around the incision |
| 4 | Patches of inflammatory cells infiltration around the incision |

| Supplementary Table S4. Histopathological score of tissue carbonization | |
| --- | --- |
| Score | Features |
| 0 | No carbonization around the incision |
| 1 | Occasional carbonization around the incision |
| 2 | Small amount of carbonization around the incision |
| 3 | Many carbonization around the incision |
| 4 | Large carbonization area around the incision |

| Supplementary Table S5. Histopathological score of coagulative necrosis | |
| --- | --- |
| Score | Features |
| 0 | No coagulative necrosis around the incision |
| 1 | Slight coagulative necrosis around the incision |
| 2 | Moderate coagulative necrosis around the incision |
| 3 | Moderate to obvious coagulative necrosis around the incision |
| 4 | Obvious coagulative necrosis around the incision |

| Supplementary Table S6. Histopathological score of incision planeness | |
| --- | --- |
| Score | Features |
| 0 | Cutting surface flattening |
| 1 | Cutting surface is slightly uneven |
| 2 | Cutting surface is uneven |
| 3 | Cutting surface is extremely uneven |
| 4 | The incision surface lose the original structure |

| Supplementary Table S7. Histopathological score of wound healing | |
| --- | --- |
| Score | Features |
| 0 | No tissue healing at the incision |
| 1 | The tissue at the incision edge is slightly hyperplastic |
| 2 | The tissue at the incision extends from the edge to the middle and is intended to heal |
| 3 | Moderate to significant healing of the tissue at the incision |
| 4 | The tissue at the incision is obviously healed |

**Figure Legends**

**Supplementary Figure S1. Structure comparison of monopolar and bipolar knife.** The figure was drawn by using software COMSOL Multiphysics(COMSOL Inc.), version 5.4, URL link: https://cn.comsol.com/company.

**Supplementary Figure S2. Polypectomy snare test model established by finite element method.** (A) Electric cutting experimental model of monopolar snare: the ground plate is attached to the lower surface of the tissue, and the snare is tightened along the convex portion of the tissue, and current flows from the electrode through the tissue to the ground electrode plate to form a circuit.(B) Simplified model of electric cutting experiment for monopolar snare. (C) Electric cutting experimental model of bipolar snare:the return electrode is attached to the upper surface of the tissue, and the snare is tightened along the raised portion of the tissue, and current flows from the electrode through the tissue to the return electrode to form a circuit .(D) Simplified model of electric cutting experiment for bipolar snare. These figures were drawn by using software COMSOL Multiphysics (COMSOL Inc.), version 5.4, URL link: https://cn.comsol.com/company.

**Supplementary Figure S3. Wounds of digestive** **tract (esophagus, stomach and colon) produced by two kinds of polypectomy snares during operation.** These pictures were captured by photo camera.

**Supplementary Figure S4. The wounds healing of esophageal, gastric and colonic mucosa on the 13^th^ day after endoscopic resection by monopolar and bipolar snare.** These pictures were captured by photo camera.


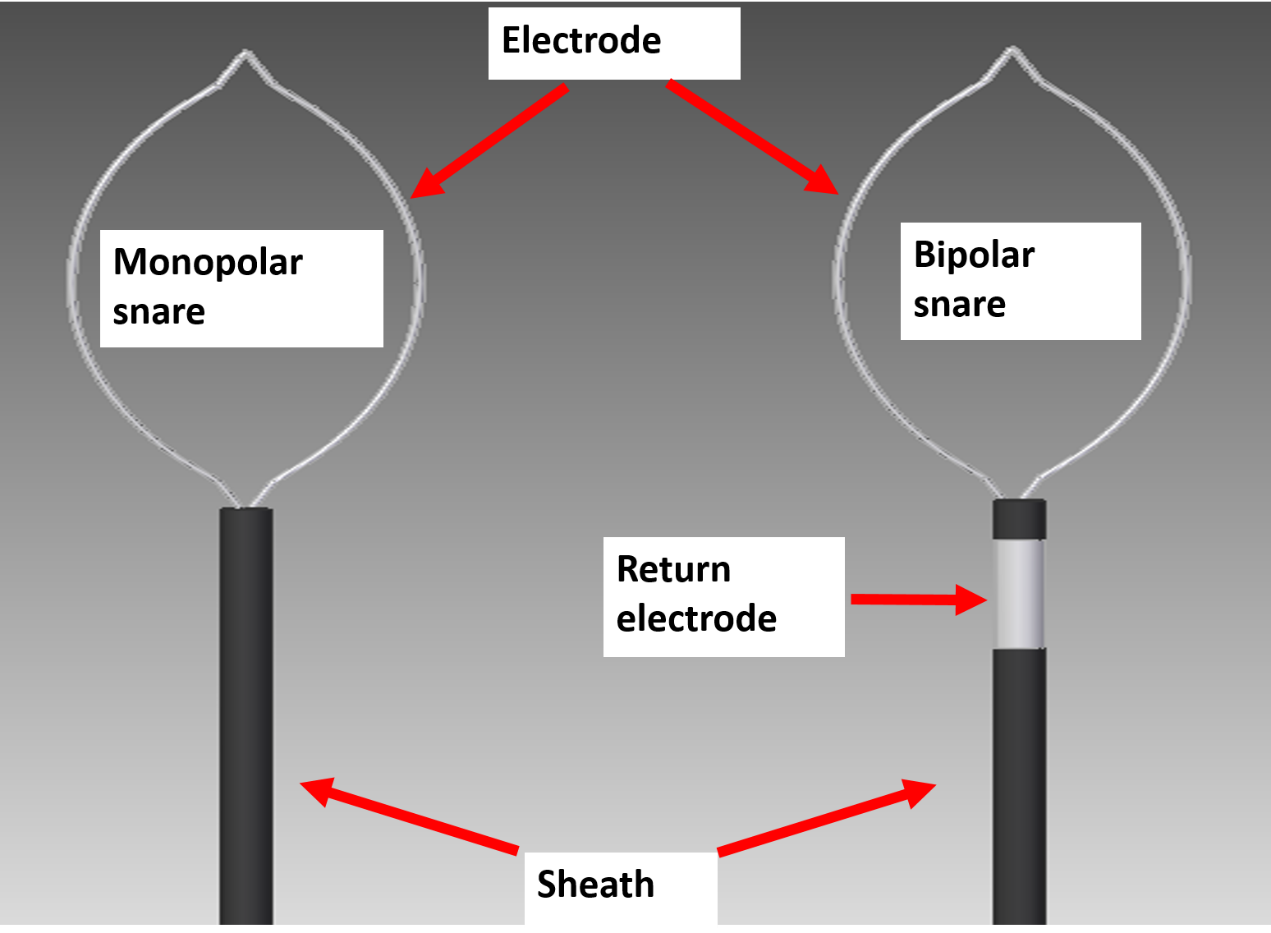


**Supplementary Figure S1**


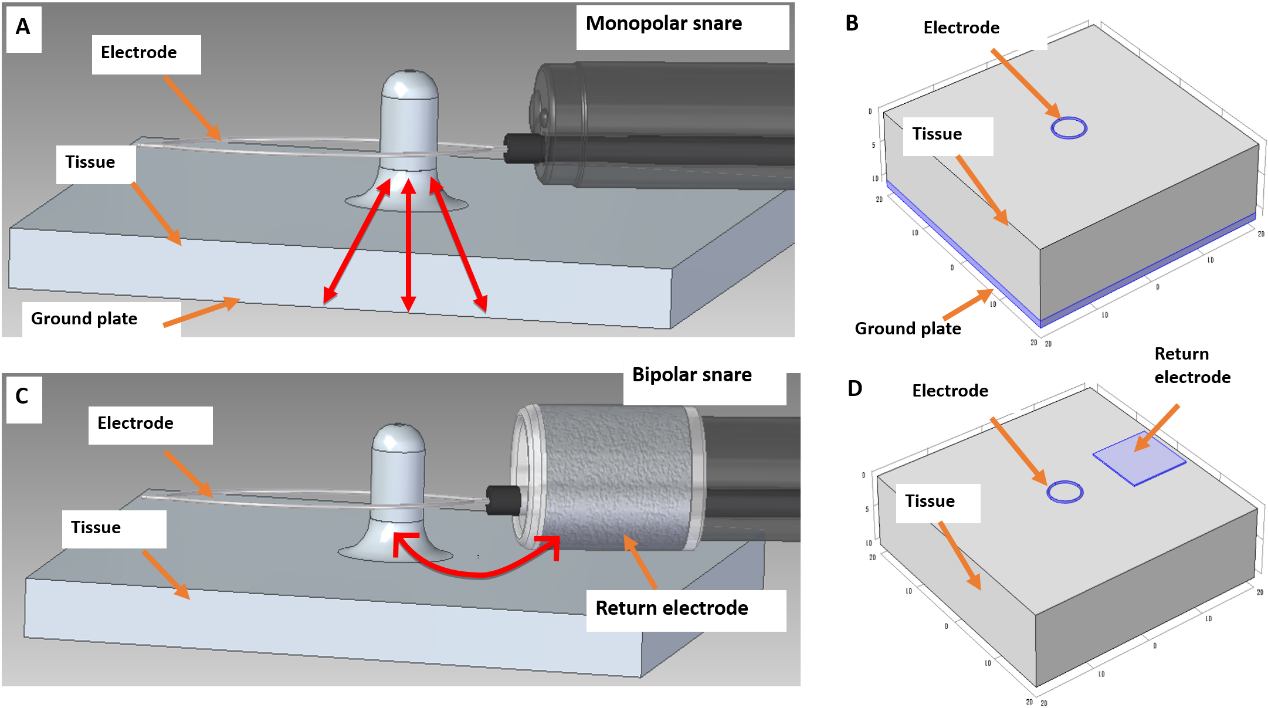


**Supplementary Figure S2**


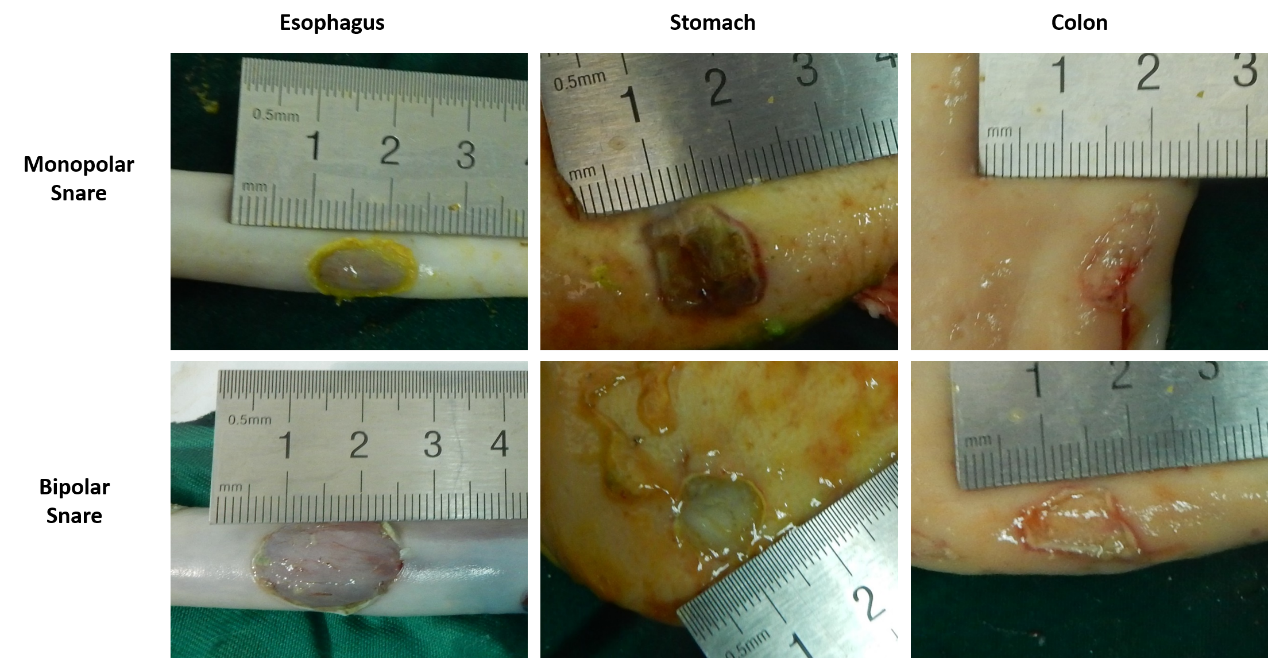


**Supplementary Figure S3**


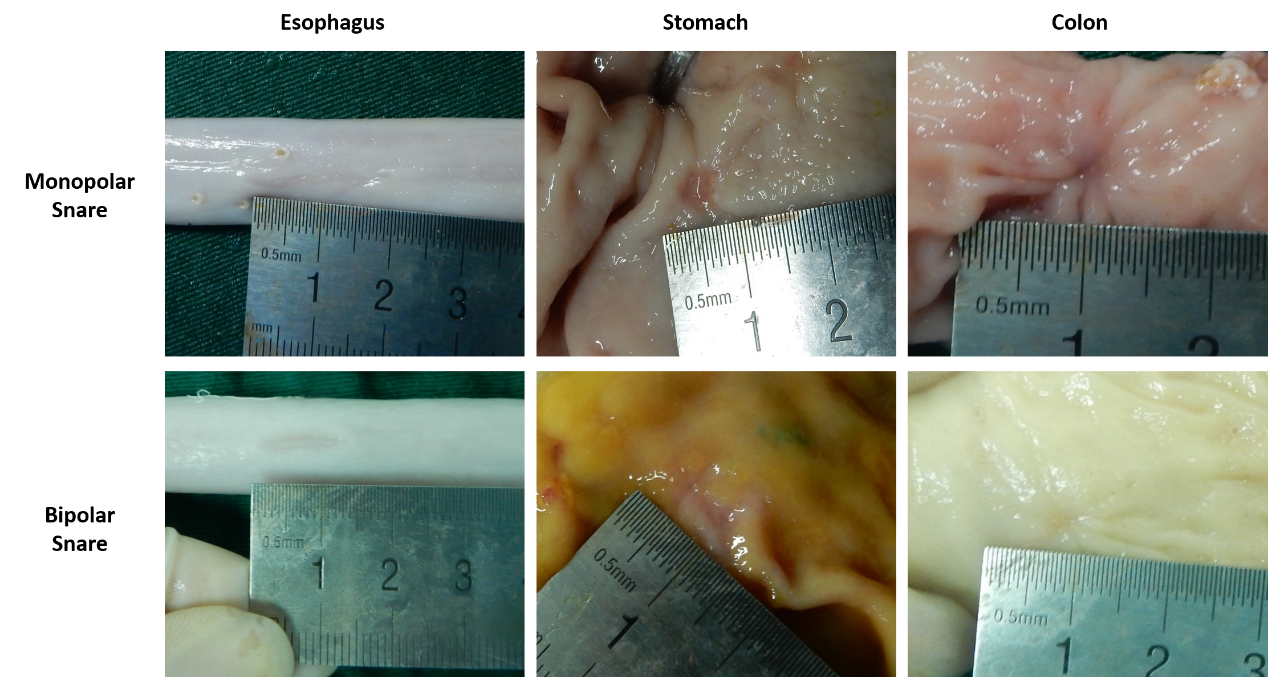


**Supplementary Figure S4**
